# Mycobacteriophages: From Petri dish to patient

**DOI:** 10.1371/journal.ppat.1010602

**Published:** 2022-07-07

**Authors:** Graham F. Hatfull

**Affiliations:** Department of Biological Sciences, University of Pittsburgh, Pittsburgh, United States of America; University of Queensland, AUSTRALIA

## Abstract

Mycobacteriophages—bacteriophages infecting *Mycobacterium* hosts—contribute substantially to our understanding of viral diversity and evolution, provide resources for advancing *Mycobacterium* genetics, are the basis of high-impact science education programs, and show considerable therapeutic potential. Over 10,000 individual mycobacteriophages have been isolated by high school and undergraduate students using the model organism *Mycobacterium smegmatis* mc^2^155 and 2,100 have been completely sequenced, giving a high-resolution view of the phages that infect a single common host strain. The phage genomes are revealed to be highly diverse and architecturally mosaic and are replete with genes of unknown function. Mycobacteriophages have provided many widely used tools for *Mycobacterium* genetics including integration-proficient vectors and recombineering systems, as well as systems for efficient delivery of reporter genes, transposons, and allelic exchange substrates. The genomic insights and engineering tools have facilitated exploration of phages for treatment of *Mycobacterium* infections, although their full therapeutic potential has yet to be realized.

## Why study phages and mycobacteriophages in particular?

Bacteriophages were discovered a little over a hundred years ago and quickly developed into workhorses for molecular biology and genetics [1]. They provided suitably sized genomes for development of early DNA sequencing techniques, although phage genomics did not really take off until the early 1990s. Mycobacteriophages—the subject of this review—became a focus of interest for addressing 5 key priorities. First, is the deep exploration of phages that infect a single common host strain (*Mycobacterium smegmatis* mc^2^155) for insights into viral diversity, evolution, and origins? Second, is the use of phage discovery and genomics as a platform for advancing science education? Third, is the investigation of all aspects of host–phage dynamics, including host range, life cycle regulation, viral defense, and counterdefense? Fourth, is the exploitation of mycobacteriophage genomes to develop tools for simplifying and advancing *Mycobacterium* genetics? Lastly, is the potential for clinical use of mycobacteriophages to control *Mycobacterium* diseases? I will provide introductory information for the general reader on the world of bacteriophages and will then discuss recent advances toward these 5 priorities.

## Introduction to the phage world

Bacteriophages are viruses that infect bacteria. They are prevalent in the environment and throughout the human body, and there are an estimated 10^31^ phage particles in total in the biosphere, making them the majority of all biological entities [2–4]. The phage population is old—plausibly originating over 3 billion years ago—and is highly dynamic. Phages are constantly infecting bacterial hosts, replicating, and replenishing the population, and there is estimated to be about 10^23^ phage infections per second, with the phage population replacing itself every few days [4].

Given these parameters, it is not surprising that phages are genomically highly diverse [5]. Phage lytic infections—those resulting in replication and production of new phage particles—kill the bacterial host, and there is strong selection for development of resistance and survival. Such mechanisms are widespread and include receptor variation, restriction-modification systems, and CRISPR-Cas arrays among a large variety of newly discovered systems [6]. Phages would not exist if they could not circumvent these defenses and resistance mechanisms, and they actively coevolve along with their hosts [7]. They do so by a variety of mechanisms including the acquisition of counterdefenses or switching to a new bacterial host that lacks the defenses. These dynamics have prominently shaped microbial evolution.

Bacteriophages can use DNA or RNA as their genetic material, which is typically encapsulated in a protein shell, sometimes with lipid components [8]. A variety of morphologies can be seen by electron microscopy, although the predominant types are those with a tail attached to a capsid (or phage head) containing double-stranded DNA (dsDNA) [9]. These dsDNA tailed phages (the *Caudovirales*) can be divided into 3 families depending on their tails: the *Myoviridae* with contractile tails; the *Siphoviridae* with long, flexible, noncontractile tails; and the *Podoviridae* with short stubby tails. The capsids are usually isometric (soccer ball-like) or prolate (elongated, rod-like) and vary in size according to the length of the DNA packaged within them. The virion genome is typically linear but may genetically be either linear or circular depending on the DNA packaging mechanism [10]. Phages using *cos*-type packaging signals have genomes with defined physical ends such that all phage particles have the same genome termini. In contrast, phages using headful DNA packaging systems have genomes that are circularly permuted and terminally redundant, with the physical genome ends different in each particle.

A key phage property is host specificity. Phage host ranges vary considerably, but it is uncommon for any given phage to infect bacteria in more than one genus. In the few instances where host range spans different genera, the bacteria are usually phylogenetically closely related [11,12]. Sometimes host range is restricted to a few strains within a species, and some phages infect only a single known bacterial strain.

Finally, phages typically can be ascribed to having one of 2 lifestyles, either lytic or temperate. The key distinction is that temperate phages can establish lysogeny within a host cell, whereas lytic phages do not. Both lytic and temperate phages can undergo cycles of lytic growth in which the phage replicates, the cell dies, and progeny phage particles are released. However, this is the sole outcome for a lytic phage (a canonical example is phage T4 of *Escherichia coli*). In contrast, temperate phages can enter one of 2 life cycles upon infection: lytic growth as described above, or lysogeny in which the lytic genes are switched off (a canonical example is phage lambda of *E*. *coli*); a prophage is established either by chromosomal integration or plasmidial extrachromosomal replication, and the lysogenic cell continues to grow. The frequency of lysogeny varies enormously depending on various parameters (e.g., growth state, multiplicity of infection), and can be as low as a few percent, or greater than 50% of infections. The prevalence of temperate phages varies depending on the host, and this is reflected in the richness of resident prophages in sequenced bacterial genomes [13]. It should be noted that some types of temperate phages do not lyse the host cell when replicating, including filamentous phages (inoviruses) such as *Pseudomonas* phage Pf [14].

## Mycobacteriophages are viruses of mycobacteria

Mycobacteriophages are phages that infect *Mycobacterium* hosts. The genus *Mycobacterium* includes several important human pathogens, including *Mycobacterium tuberculosis*, the causative agent of tuberculosis, and *Mycobacterium avium* complex (MAC) and *Mycobacterium abscessus* complex (MAB) that are notable opportunistic pathogens. Mycobacteria characteristically are acid-fast staining and have unusual cell walls containing mycolic acids, glycolipids, and glycopeptidolipids (GPLs) surrounding the cytoplasmic membrane and peptidoglycan layer. This unusual cell wall presents a unique challenge to bacteriophages that must bind specifically, navigate their tail tips to the membrane, and inject their DNA. At the conclusion of lytic growth, they must also compromise these walls to release progeny phage particles from the cell.

The first mycobacteriophages were isolated in the 1950s—using *M*. *smegmatis* as a host—primarily for phage-typing mycobacterial infections, taking advantage of mycobacteriophage host range variations [15]. Some of these typing phages have been recovered, although others are lost [16,17]. The first mycobacteriophage genome to be fully sequenced was that of L5 [18], which was originally isolated in the 1950s in Japan [19]. This was followed by reports of genome sequences of phages D29, TM4, and Bxb1 [20–22], but further exploration of mycobacteriophage diversity would require additional phage isolation, most prominently using *M*. *smegmatis* mc^2^155 as a host [23].

### Phage discovery and genomics as integrated research-education programs

With just a handful of sequenced mycobacteriophages available, in the early 2000s it was clear that gaining a fuller understanding of viral diversity would need a deeper dive into phage isolation and genomic analyses [24]. An alternative approach—metagenomic analysis of enriched viral samples—is also attractive, but generally provides informative data sets rather than individual phages that can be propagated, engineered, investigated mechanistically, and potentially used translationally. Envisaging the need to isolate phages at substantial scale, we developed integrated research-education programs in which phage discovery and genomics is the primary goal, providing research opportunities to young scientists including high school students and first year undergraduate students [25]. The Phage Hunters Integrating Research and Education (PHIRE) starting in 2002 provided research experiences locally in Pittsburgh [26,27] with the platform expanding into the Science Education Alliance Phage Hunters Advancing Genomics and Evolutionary Science (SEA-PHAGES) in 2008 [28,29]; both programs are supported by the Howard Hughes Medical Institute (HHMI). The SEA-PHAGES program has grown with addition of institutions from the United States and beyond, and now has over 170 participating schools with over 5,500 students each year. SEA-PHAGES is an example of an Inclusive Research Education Community (iREC) that encompasses institutions ranging from community colleges to Research I universities and involves all students without pre-selection based on generally irrelevant criteria [29,30].

**Fig 1:**
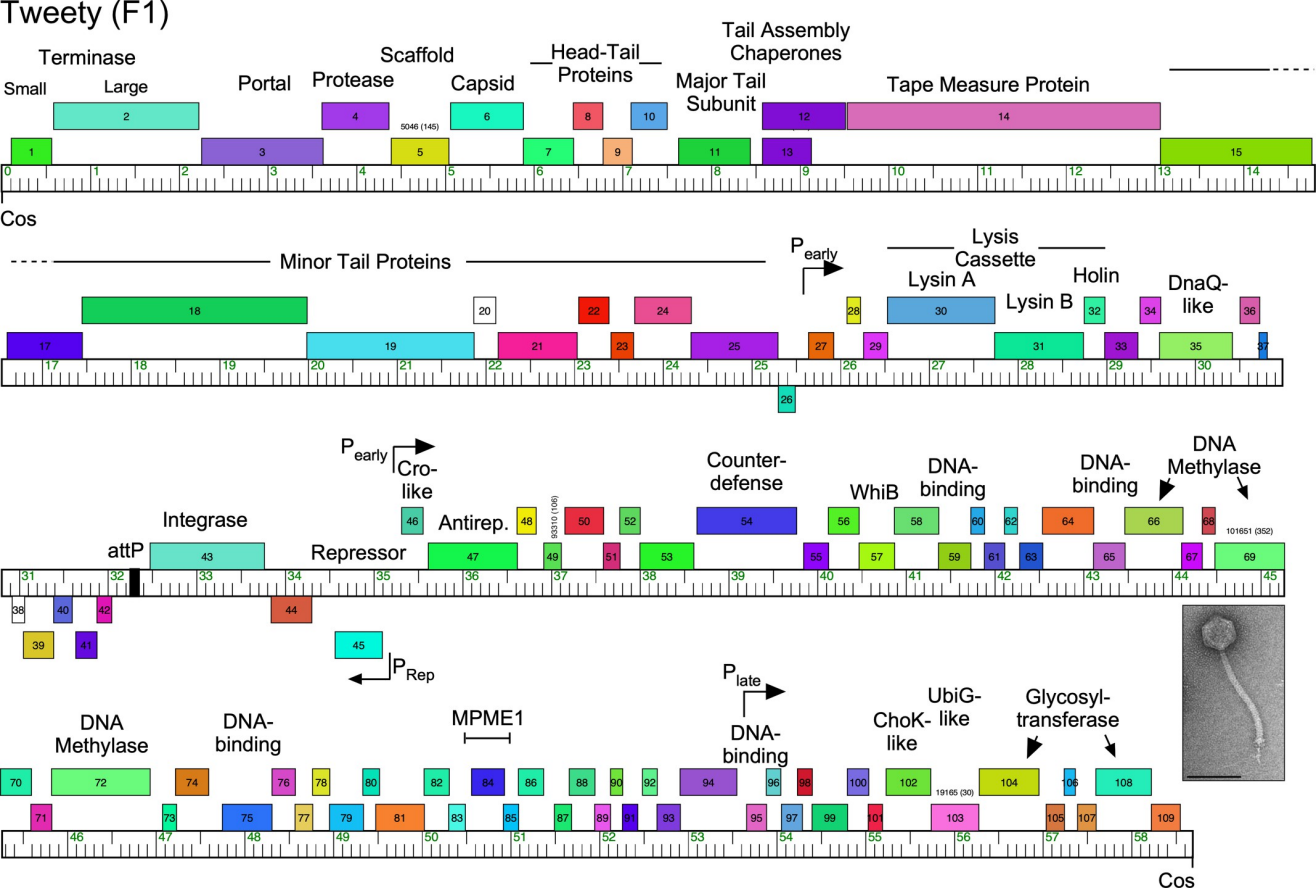
Genome organization of Mycobacteriophage Tweety. Phage Tweety is a member of Subcluster F1, is temperate, has a siphoviral morphology (inset, electron micropraph; scale marker, 100 nm), and its genome organization illustrates several common features. The genome is represented as a bar with markers every 1 kbp, and the predicted genes are shown as colored boxes above or below the genome, indicating rightwards- and leftwards-transcription, respectively. Gene numbers are shown within the boxes and predicted functions are shown above. The virion structure and assembly genes are arranged in a rightwards-transcribed operon at the left end of the genome (genes 1 to 25), followed by the lysis cassette. The repressor (*45*) and putative Cro-like (*46*) genes are divergently transcribed, and the integration cassette (*attP* and *43*) are located nearby, with *attP* defining a “left arm” from *cos*–*attP* and a “right arm” from *attP–cos*. Note that the left arm genes—predominantly the virion structure and lysis genes—are relatively large and have known functions, whereas the right arm genes are relatively small, and most have unknown functions. Shown are the positions of the MPME mobile element and the counterdefense gene, *54*. The positions of putative early and later lytic promoters as determined by RNAseq are indicated. Note that genes are arranged in long operons such that there are relatively few transcriptional changes (tdc), such as at the *25*/*26* and *45*/*46* junctions.

These programs have produced a massive archive of individual bacteriophages and a rich data set of sequenced and manually annotated genomes focusing on carefully chosen bacterial hosts. The most widely used host is *M*. *smegmatis* mc^2^155, a natural extension from earlier studies; over 10,000 individual phages have been isolated, by far the deepest investigation of phages using a single bacterial strain. Over 2,000 have been sequenced, and sampling of the remainder suggests that no substantial component of the diversity has been missed. An additional 10,000 phages have been isolated using other bacterial genera within the phylum *Actinobacteria*, including, *Arthrobacter*, *Corynebacterium*, *Gordonia*, *Microbacterium*, *Rhodoccocus*, and *Streptomyces* [31]. Information about the entire phage collection is available at http://phagesdb.org. A genome map of mycobacteriophage Tweety [32] is shown as an example in Fig 1 and illustrates several of the features described below.

### Mycobacteriophage genomics, diversity, and architecture

What have we learned about mycobacteriophage genomes? Several key themes have emerged. First, the overall diversity is impressive, although complex in its structure. Comparative genomics clearly shows that some phages are closely related, and a system was established early on to accommodate these relationships. Phages that are closely related to each other are grouped into “clusters,” with different phages in different clusters (e.g., Cluster A, Cluster B, Cluster C). Many clusters have recognizable divisions, as seen through average nucleotide identity (ANI) comparisons, and are thus divided into “subclusters” (e.g., Subcluster A1, Subcluster A2, Subcluster A3). Phages for which there are no close relatives are recognized as “singletons”. The initial parameters used for cluster grouping required recognizable nucleotide sequence similarity spanning greater than 50% of the genome lengths, and with only a few dozen genomes all mycobacteriophages could be grouped without any evident challenge to these thresholds [33–35].

**Fig 2:**
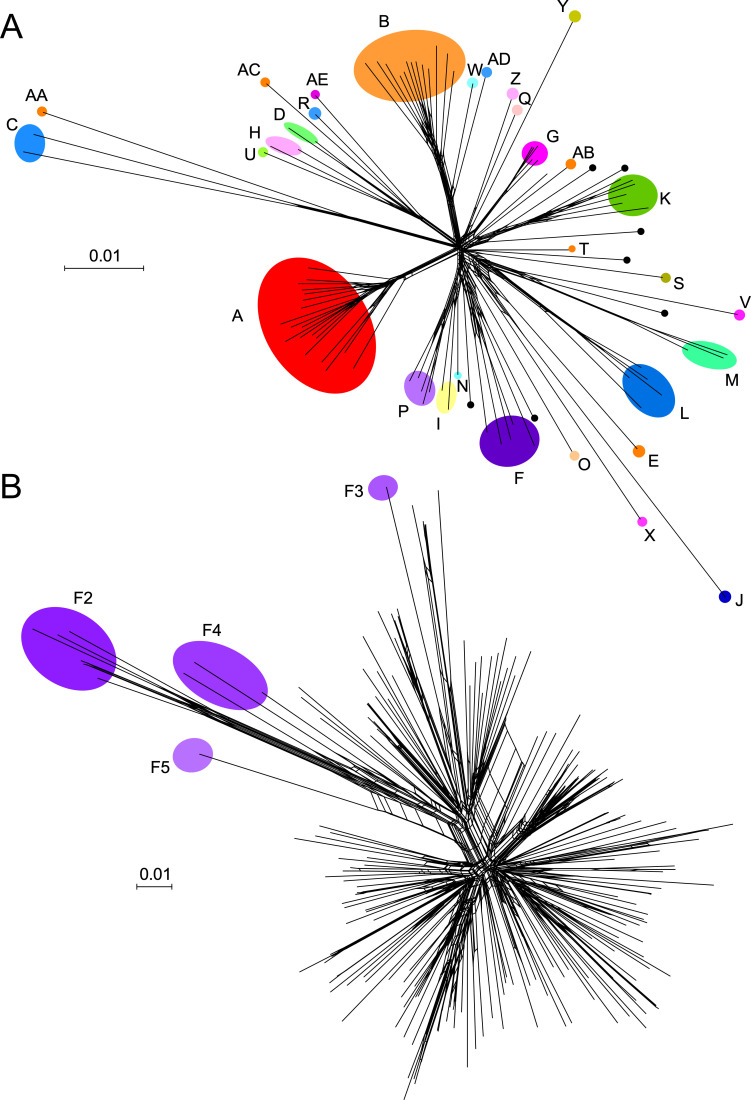
Diversity of mycobacteriophage genomes displayed as network phylogenies based on shared gene content. (**A**) Relationships among representative members of clusters, subclusters, and singleton genomes. One member of each mycobacteriophage cluster and subcluster together with the 7 singletons were compared using Splitstree [39] with a nexus file recording the numbers of shared genes. Clusters are illustrated with colored shading; note that some clusters (e.g., Cluster A) contain several subclusters indicated as nodes, whereas other clusters are not subdivided. Singletons are shown as unlabeled black circles. (**B**) Diversity of Cluster F mycobacteriophages. All currently sequenced Cluster F mycobacteriophages (*n* = 188) are displayed as nodes in a network phylogeny using Splitstree. Colored circles show the positions of the Subclusters F2 to F5 genomes; all of the others (*n* = 177) are grouped in Subcluster F1. This illustrates the substantial intracluster diversity, and pairwise comparisons of Subcluster F1 phages show they may share as few as 40% of their genes.

As the mycobacteriophage collection grew to several hundred genomes, these “boundaries” of similarity between clusters became fuzzy, with the discovery of phages that only just meet or just miss the thresholds for inclusion in a cluster, or are close to the threshold for inclusion in more than one cluster [36]. This suggested that the cluster boundaries are artificial and do not reflect hard biological barriers to recombination and exchange of information between phage genomes. Phages therefore appear to form a continuum of diversity but with unequal representation and heterogenous sampling [36]. This heterogeneity is reflected in the abundance of more than 700 Cluster A phages and more than 350 Cluster B phages, compared to the 7 singletons and 10 clusters with 5 or fewer phage members. As the phage collection continued to expand, the thresholds were redefined with clearer and more quantifiable parameters, and we currently use a threshold of 35% shared gene content (SGC) for cluster inclusion [37]. This is determined by assorting all of the gene products into groups of related proteins (“phamilies,” or “phams”) using the program Phamerator [38] and a pipeline based on MMseqs2. This can then be used to determine the number of shared phams, and a tool is available at https://phagesdb.org/ for simple pairwise SGC comparisons. The overall genomic relationships among the mycobacteriophages can be viewed as a network phylogeny using Splitstree [39], simplified by using a single representative of each cluster and subcluster together with the 7 singletons (Fig 2A). There are numerous examples in which 2 genomes from different clusters (e.g., Bxb1 from Subcluster A1 and Bipper from Cluster Y) have no shared genes at all. There are also examples of phages in different clusters sharing considerable number of genes (e.g., 25% between Fruitloop and Omega in Clusters F and J, respectively). Not surprisingly, there is also considerable intracluster diversity illustrated by the example of the Cluster F phages (Fig 2B). There are currently 5 F subclusters (F1 to F5), with by far the vast majority (95%) grouped in Subcluster F1 (Fig 2B). Pairwise comparisons show that genomes within Subcluster F1 can share as few as 40% of their genes. In general, the cluster/subcluster/singleton “taxonomy” is of pragmatic use—because of other shared properties—but it is a crude representation of phage diversity.

The second key finding is that phage genomes are architecturally mosaic. Each genome is essentially a compilation of modules or mosaic “tiles” that can be assembled in vast numbers of different combinations (see Fig 1) [23]. These modules are often single genes, and amino acid sequence comparisons reveal many instances of genomes having a shared gene but in different genomic contexts, such that the flanking genes are different. Most commonly, there is a lack of nucleotide sequence similarity and only weak amino acid sequence identity, reflecting relatively old recombination events in phage evolution. The mechanisms giving rise to genome mosaicism are not completely clear, but likely involve non-sequence-directed illegitimate recombination events between genomes [3,23]. This is supported by observations of mosaic boundaries between genomes where there is extensive sequence similarity, and the finding that these usually occur at gene—and sometimes domain—boundaries [3,4]. Mobile elements such as transposons and homing endonucleases are likely also involved, as well as integrase-mediated site-specific recombination events [40,41]. We note that several transposons have been noted in the mycobacteriophage genomes, including the novel small mycobacteriophage mobile elements (MPMEs) first described in Cluster G genomes [42] (see Fig 1). Phage-encoded homologous recombination systems that can act on short segments of imperfect homology are also implicated in events that can give rise to mosaicism [43].

The third key finding is that there are clearly architectural constraints on phage genomes. In general, phage average gene size is smaller (approximately 0.6 kbp) than in bacterial genomes (approximately 1 kbp) [33], and the genes are assembled into longer operons than in bacteria, so that there are fewer changes in the direction of transcription (tdc) (see Fig 1). The vast majority of mycobacteriophages (in total, and as defined by clusters/subclusters/singletons) have siphoviral morphologies, and these all contain a characteristic operon (approximately typically 20 to 25 kbp) of virion structure and assembly genes (Fig 1). These genes are on average more similar in length to bacterial genes. In contrast, the nonstructural genes in the rest of the genome are much smaller on average, so that there is a bimodal distribution of phage gene length (Fig 1). Genome length varies substantially, and mycobacteriophages with siphoviral morphologies vary from approximately 40 kbp to approximately 110 kbp. Because the genome requirements for virion structure and assembly genes don’t vary greatly, it is the number of nonstructural genes that varies as genome length changes. Some of these genes have predicted functions such as in DNA replication and metabolism, but most are of unknown function (Fig 1). The total number of genes of unknown function among the 2,000 sequenced mycobacteriophages is impressive (>100,000) and suggests that the phage population harbors the greatest reservoir of unexplored gene diversity and function.

### Mycobacteriophage lifestyles

*M*. *smegmatis* mc^2^155 is prophage-free, but a majority of the mycobacteriophages isolated on this strain are temperate. Specifically, of the 31 clusters and 7 singletons, 21 are temperate or have temperate representatives (Clusters A, E, F, G, I, J, K, L, M, N, P, Q, T, X, Y, Z, and singletons IdentityCrisis, Kumao, LilSpotty, MalagasyRose, and Sparky). It is noteworthy that it is relatively common to isolate phages that have clear plaque morphologies and appear obligatorily lytic but are members of a cluster where most other members are temperate. An early example of this was phage D29 [20], which is grouped in Subcluster A2 along with temperate phages such as L5 [18], but has a deletion that removes the repressor gene [20,44]. There are many similar examples in other clusters.

The temperate phages typically encode a repressor that is required for the maintenance of lysogeny (analogous to lambda cI), and an integrase that mediates site-specific integration into the bacterial genome to form a prophage. It is noteworthy though that several phages—all within Cluster A—form extrachromosomal prophage replicons (i.e., plasmidial) and code for *parAB* partitioning systems instead of an integrase [45]. For many mycobacteriophages (with the notable exception of Cluster A), the repressor gene is centrally located and transcribed divergently from the early lytic genes, with the repressor’s most proximal gene being Cro-like (i.e., analogous to phage lambda Cro). Typically, both repressor and Cro-like proteins contain predicted DNA-binding domains and their genes are separated by an intergenic region of 100 to 300 bp containing promoters for early lytic and repressor gene expression, and an operator site for repressor binding [46,47] (see Fig 1). However, few of these repressor or Cro-like proteins have been studied in detail. Two unusual immunity systems warrant further discussion.

### Integration-dependent immunity: An atypical system for lysogenic establishment

**Fig 3:**
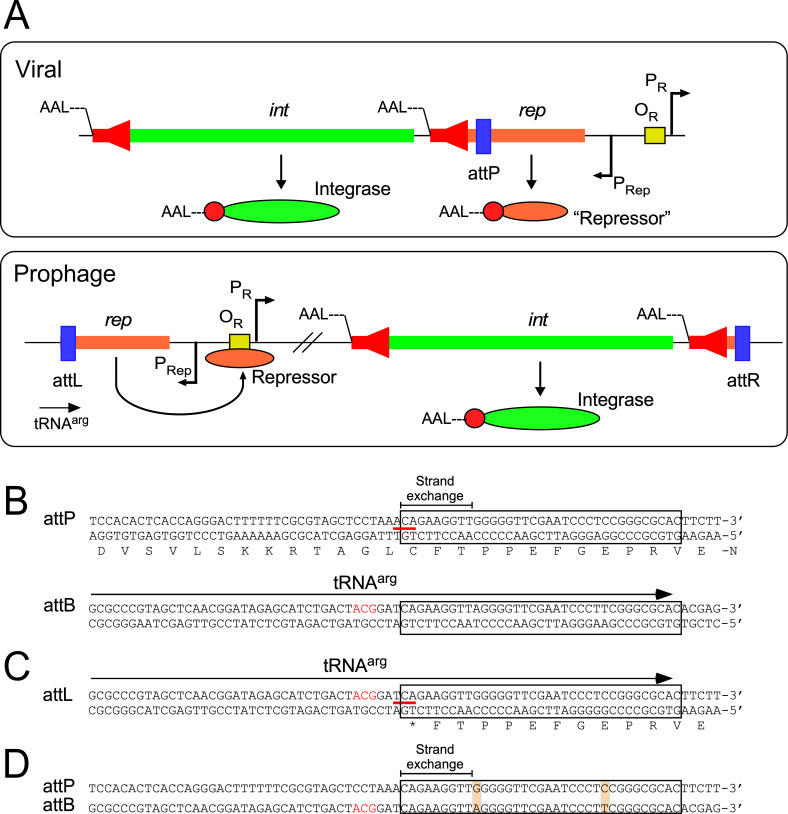
Integration-dependent superinfection immunity systems. (A) Typical organization of the immunity regions of phages encoding integration-dependent immunity systems. In the viral genome, the repressor (*rep*) and integrase (*int*) genes are cotranscribed from the P_Rep_ promoter, and the phage attachment site (*attP*, blue box) is located within the repressor gene. The virally encoded Integrase and Repressor proteins both carry C-terminal ssrA-like tags (——LAA, or similar) targeting the proteins for proteolytic degradation (red arrows in the int and rep genes; red circles in the proteins; the N-termini are indicated). The virally encoded form of the Repressor is not active in conferring superinfection immunity. The establishment of lysogeny requires integrase-mediated site-specific recombination between the phage *attP* site and a chromosomal *attB* site (which overlaps a host tRNA gene) to form a prophage. Integrative site-specific recombination removes the ssrA-like tag from the repressor and the stable, active form of the repressor binds to the operator (O_R_, yellow box) to shut down the early lytic promoter (P_R_) and confer superinfection immunity. (**B**) Organization of the *attP* and *attB* sites of phage BPs and *M*. *smegmatis*. Both DNA strands are shown, and the amino acid sequence of part of the leftwards-transcribed repressor is shown; DNA and protein polarities are indicated. The 35-bp common core sequence (conserved in *attP*, *attB*, *attL*, and *attR*) is boxed, and the region within which strand exchange for recombination must occur is indicated. The codon spanning the left side of the common core is indicated by a red line, and the third position base is shown in bold pink type. The location of the tRNA^arg^ gene at *attB* is shown by an arrow; the anticodon is shown in red type. (**C**) Organization of the BPs *attL* site. Conservation of the common sequence between *attP* and *attB* results in construction of an active tRNA^arg^ gene when the prophage is established. However, at *attL* the repressor gene encounters a termination codon (TGA) formed at the junction of the bacterial and phage sequences, changing the third base of a cysteine codon (red line) to a nonsense codon; the third position base is shown in bold pink type. (**D**) Alignment of *attP* and *attB* showing the common core (boxed), and the positions of 2 mismatches in the common core, neither of which introduced mispairing in the tRNA^arg^ product. Only the top strand of each site is shown.

First, several mycobacteriophages (including those in Clusters G, N, and P) use an unusual integration-dependent immunity system (Fig 3) [46]. In the canonical phage lambda system, establishment of superinfection immunity—prophage repressor expression that prevents superinfection—is distinct from chromosomal integration [48]. Both are under regulatory control by the cII protein but are separable molecular events. That is, establishment of superinfection immunity (protection from reinfection by particles of the same phage) does not require integration, and integration is not required to establish immunity. In contrast, the integration-dependent immunity systems fundamentally operate differently, and there are several distinguishing and unusual features [46]. First, the repressor and integrase genes are cotranscribed in an operon, and the attachment site (*attP*) used for integrase-mediated site-specific recombination is within the repressor gene itself (Fig 3A). This is seemingly paradoxical because integration and prophage formation leads to loss of the 3′ end of the repressor gene, and yet it is the prophage-expressed form of the repressor that needs to be active to confer immunity (Fig 3A). The resolution of this conundrum is that the virally encoded form of the repressor contains a C-terminal ssrA-like tag that targets it for proteolytic degradation. Integration leads to a C-terminally truncated repressor protein that is stable because of loss of the proteolytic tag, and thus active for superinfection immunity (Fig 3A). However, this leads to another difficulty. Lysogeny is usually established in only a small proportion of infections (5% to 20%, depending on conditions), and if integration always results in immunity, what prevents all infections from entering lysogeny? The answer is that the integrase protein itself is also under proteolytic control, and similarly carries a C-terminal ssrA-like tag (Fig 3A). The frequency of lysogeny is therefore likely determined by the activity of host proteases, analogous to the way *E*. *coli* FtsH determines the activity of lambda cII [49].

Like many phages that use integrases of the tyrosine family of site-specific recombinases (i.e., “tyrosine integrases”), all of the phages using integration-dependent immunity systems use an *attB* site overlapping a host tRNA gene (Fig 3B). The common core sequence—a 35-bp region shared by *attP* and *attB*—must therefore contain not only the sequences for integrase binding and strand cleavage, but also the 3′ end of the tRNA so that tRNA function is maintained in the lysogen (Fig 3B). Amazingly, the sequences for BPs integration at *attL* not only result in loss of the C-terminal ssrA-like tag from the repressor, but also the bacterium genome sequence base pair immediately adjacent to the common core changes the third position of a cysteine codon in the repressor gene and introduces a translation termination codon (Fig 3C). In some instances, such as with phage BPs, there are mismatches between the common cores in *attP* and *attB*, although these do not impair normal base paring in the tRNA (Fig 3D).

Finally, these integration-dependent immunity systems do not evidently code for a recombination directionality factor (RDF) that normally controls the ratio of integrative and excisive integrase-mediated recombination events [50]. As such, the frequency of these may simply be determined by the amount of stable integrase in the cell. A consequence of this is that the attachment sites (*attP*, *attL*, *attR*) do not require arm-type integrase binding sites, and only use the common core sequences around the scissile bonds for integrase binding (Fig 3). It is easy to imagine how this evolved, because *attP* lies within the repressor gene, and an *attP* site the size of that for lambda (250 bp), would impose severe constraints on the amino acid sequence of the repressor (Fig 3). These types of immunity systems are not only found in mycobacteriophages, but also in a variety of the diverse prophages resident in sequenced *Mycobacterium* genomes [51].

### The repressor–stoperator system of Cluster A mycobacteriophages

The second system of interest is the repressor–stoperator systems of Cluster A phages. Bioinformatic prediction of repressor genes can be challenging, and the repressors of Cluster A genomes (e.g., L5, Bxb1) were identified using genetic and biochemical methods [52,53]. Moreover, the Cluster A repressor genes are situated toward the right end of the genome within a large set of leftwards-transcribed genes and are not closely linked to the centrally located integrase gene. The Cluster A genomes unusually possess many asymmetric 13 to 14 bp sequence motifs located throughout the genome to which the repressor binds [54,55]. One of these is a true operator site and overlaps the early lytic promoter, P_left_. The others are referred to as “stoperators,” as they are proposed to be sites where repressor binding results in termination of transcription, rather than inhibition of transcription initiation [55]. The sites are located primarily within short intergenic regions, are in 1 orientation with respect to the direction of transcription, and confer repressor-dependent orientation-specific down-regulation of a reporter gene [55]. The presumed function is to silence phage genes in lysogeny and prevent leaky expression that could impose a growth disadvantage to the lysogen [55]. The immune specificities of the numerous Cluster A phages are highly complex but reveal insights into how these specificities have evolved [54].

RNAseq approaches show that many mycobacteriophages display 2 main patterns of lytic gene expression, occurring early (approximately 30 min) and late (approximately 120 min after infection [44,56–58]). Early lytic promoters are often SigA-like, with recognizable −35 and −10 hexamers recognized by the host RNA polymerase and operator sites for repressor binding [46,55,59]. Few late promoters have been characterized, but transcription starts upstream of the virion structure and assembly genes sometimes upstream of the right *cos*-site (Fig 1). Further investigation of the phage late promoters and their regulation is warranted as they are highly active and potentially useful for heterologous gene expression.

### Phage integration systems

**Fig 4:**
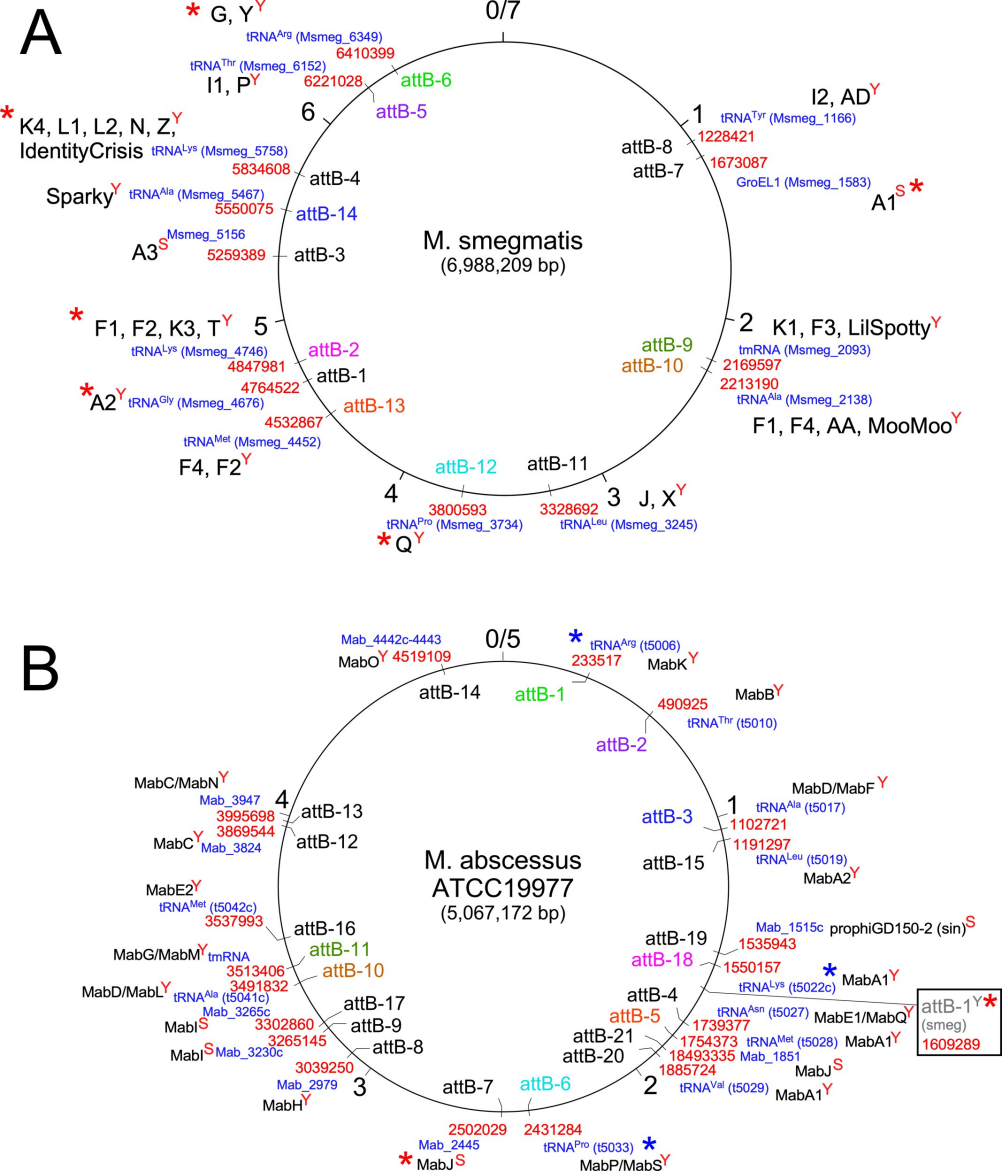
Chromosomal *attB* attachment sites used by temperate phages in *M*. *smegmatis* and *M*. *abscessus*. (**A**) *attB* sites in the *M*. *smegmatis* mc^2^155 genome. The 7-Mbp *M*. *smegmatis* genome is represented as a circle with markers at each kbp indicated. The *attB* sites (e.g., attB-1, attB-2, etc.) are shown, together with the genome coordinates in red type; the genes are shown in blue type. The font color of the *M*. *smegmatis attB* sites (i.e., attB-x^smeg^) label is coordinated with homologous sites in *M*. *abscessus* (i.e., attB-x^Mab^) shown in panel B. The names of clusters or subclusters (or the phage name if it is a singleton) within which one or more phages use that site for integration are shown in large black type. “Y” or “S” superscripts on the cluster names denote whether the site is used by tyrosine- (Y) or serine (S) -integrases. *attB* sites for which integration-proficient vectors have been developed are indicated with a red asterisk. (**B**) *attB* sites in the *M*. *abscessus* ATCC19977 genome mapped by identification of integrated prophages. The font color of the *attB* sites (i.e., attB-x^Mab^) is coordinated with their homologues in *M*. *smegmatis* (i.e., attB-x^smeg^) shown in panel A. Genome coordinates are shown in red type; genes are shown in blue type. The clusters/subclusters of prophages for which members are found integrated in those sites are shown in black type. “Y” or “S” superscripts on the cluster names denote whether the site is used by tyrosine- (Y) or serine (S) -integrases. The integration vectors based on phage L5 (attB-1^smeg^) integrate at a homologous site in *M*. *abscessus* (shown in box) although no prophages have been identified there. This and the attB-7^Mab^ site are only ones for which integration vectors have been shown to work (marked with red asterisks), but several vectors developed for *M*. *smegmatis* are predicted to also work in *M*. *abscessus* (blue asterisks).

Most of the temperate mycobacteriophages encode integration systems similar to those described above for the integration-dependent immunity systems. The integrases can be either lambda-like tyrosine integrases (Int-Y) or large serine-integrases (Int-S), although the former are more common (see Fig 1). At least 14 different *attB* sites have been identified (Fig 4A), distributed around the *M*. *smegmatis* genome. Interestingly, the Int-Y genomes exclusively use *attB* sites overlapping host tRNA genes, and the common core facilitates reconstruction of the tRNA upon integration. Because these common cores are 25 bp or longer, the *attB* site for most Int-Y phages can be predicted using homology searches. This is not so for Int-S phages, for which the common core can be very small (as few as 3 bp), and they do not integrate into tRNA genes [60–63]. Their *attB* sites therefore have to be determined experimentally, and Bxb1 and Bxz2 (Subcluster A1 and A3, integrating into attB-7 and attB-4, respectively; Fig 4A) have been shown to integrate within the protein-coding genes for *groEL1* (Msmeg_1583) and a putative DNA glycosylase (Msmeg_5156), respectively [61,64]. Interestingly, Bxb1 integration inactivates GroEL1 and confers a defect in biofilm formation [65].

Interestingly, although *M*. *tuberculosis* genomes are devoid of full-length prophages, prophages are abundant and diverse in *M*. *abscessus* genomes [51,66], and the *attB* sites can be easily determined (Fig 4B). Of the 21 *attB* sites, 16 are used by Int-Y and 5 by Int-S (Fig 4B). Interestingly, of the 16 Int-Y *attB* sites, only 12 overlap a host tRNA gene and 4 are either within reading frames or in regulatory sequences. Eight of the *attB* sites used by Int-Y are conserved between *M*. *abscessus* and *M*. *smegmatis* (Fig 4). Ten of the *attB* sites identified in *M*. *smegmatis* are also present in *M*. *tuberculosis* H37Rv [67].

### Host range

The bacterium-phage billion-year-old battle for supremacy has profoundly influenced microbial evolution, sharply honed the variation among bacterial and phage strains, and molded the specificity of phages for their hosts. The expectation is thus that phages isolated on one host may infect other hosts, but that there will be substantial variations in host range. Furthermore, host range is malleable, and if an appropriate nonpermissive strain is tested, host range mutants (HRMs) of the phage can be readily isolated that either expand or switch the phage preferences [68].

Currently, our understanding of the host range of mycobacteriophages is at low resolution, with relatively few *Mycobacterium* strains tested [68–70]. There is a great deal more to be learned by expanding the scale of experimental analyses, and determination of the phage preferences for hundreds of *Mycobacterium* species and strains. However, important insights have been gleaned by determining the phage infection profiles on *M*. *tuberculosis* strains, *M*. *abscessus* clinical isolates, and other *M*. *smegmatis* strains [68,70,71]. A notable finding is that there is a close correlation between the ability of phages to infect both *M*. *smegmatis* mc^2^155 and *M*. *tuberculosis* H37Rv and their cluster/subcluster/singleton designation [68]. The correlation is imperfect in that not all phages within a cluster/subcluster necessarily infect both strains, but there are many groups for which none of the phages infect both strains. Interestingly, a phage isolated in the 1960s (DS6A) only infects strains in the *M*. *tuberculosis* complex [72].

Early host range studies with *M*. *tuberculosis* used the common H37Rv lab strain [68]. In general, phages isolated on *M*. *smegmatis* that efficiently infect *M*. *tuberculosis* H37Rv also infect other *M*. *tuberculosis* strains [71]. There are some exceptions though, and phage Muddy efficiently infects only about one-third of the *M*. *tuberculosis* reference strains tested [71]. However, HRMs of Muddy can be isolated on the “nonpermissive” strains with a single amino acid substitution in the putative tail spike protein that not only efficiently infect the strain from which it was recovered, but also all *M*. *tuberculosis* (lineage 1 to 4) strains [71]. Interestingly, phage BPs does not efficiently infect *M*. *tuberculosis* H37Rv, but HRMs can be readily isolated that do, and these also have substitutions in a putative tail spike protein [42,68]. However, none of the HRMs tested infect any of the other *M*. *tuberculosis* reference strains [71]. Presumably, different HRMs could be isolated on other nonpermissive *M*. *tuberculosis* isolates.

*M*. *abscessus* clinical isolates are at the opposite end of the spectrum to *M*. *tuberculosis* in their variation in phage infection profiles [70]. In general, *M*. *abscessus* isolates have either smooth or rough colony morphotypes, with the smooth strains having abundant surface GPLs; relatively few of the *M*. *smegmatis* phages infect and kill any of the smooth isolates [70]. The phages that infect *M*. *abscessus* rough colony strains are generally within clusters/subclusters that also infect *M*. *tuberculosis* [70]. However, there is great variation among *M*. *abscessus* clinical isolates in their phage infection profiles, even among those that overall are very closely related genetically [70]. It would thus be misleading to test just a single isolate of *M*. *abscessus* and assume that its phage infection profile was representative of all isolates.

What determines phage host range? There are a multitude of players, but they can be generally grouped into those acting at the cell surface (e.g., receptors) and those that act after the DNA has entered the cell (e.g., restriction). Interestingly, many *Mycobacterium* strains—including *M*. *abscessus*—are CRISPR-free that eliminates a major potential contributor to host range, but they likely have a variety of restriction–modification (R–M) systems [73–76]. They may also vary in their surface structures that are recognized as receptors by the phages, although few phage receptors in *Mycobacterium* strains have been described. Curiously, GPLs are implicated as potential receptors for phage I3 infection of *M*. *smegmatis*, and yet their abundance in smooth *M*. *abscessus* strains is implicated in reduced phage infection [77]. As noted above, several phage HRMs have been described with altered tail spike proteins and these presumably alter receptor recognition by the phages, even though the receptors are not well defined. But some HRMs map in other genes including in portal proteins likely responding to defense mechanisms operating at post-injection phage growth [78].

### Prophage-mediated viral defense systems

Interestingly, prophages can be a major source of phage defense systems [57,79,80]. It is no surprise that lysogens are immune to superinfection by the same (and closely related) phages (i.e., homotypic defense), and the mechanisms of repressor-mediated immunity have been long studied. However, prophages can play more complex roles by expressing genes that influence the infection of other, unrelated phages (i.e., heterotypic defense) [79,81]. In some instances, these heterotypic defenses can be very specific for one or only a small number of superinfecting phages, while having no effect on genetic siblings or cousins of those phages. Because *M*. *abscessus* genomes are so richly endowed with prophages (see Fig 4B), these are prime candidates for influencing the highly varied phage infection profiles [51].

Many different prophage-mediated defense systems have been described, some of which likely act by influencing adsorption or DNA injection for the superinfecting phage, and others that prevent phage infection through abortive infection systems. One group of phages prominently implicated in these systems are the Cluster N mycobacteriophages, which are genomically diverse in a central portion of the genomes—adjacent to the immunity and integration region—and each phage has 3 to 9 genes that are expressed lysogenically [79]. In one example, an *M*. *smegmatis* lysogen carrying a Charlie prophage expresses a protein, gp32, a predicted membrane protein, and defends against infection by phage Che9c (Cluster I1). Charlie gp32 not only impairs plaque formation but also lysogeny, and gp32 likely acts in an exclusion-like process, preventing DNA injection into the cytoplasm [79]. Che9c is the only known phage to which Charlie gp32 provides defense. In a second example, phage Phrann codes for a putative (p)ppGpp synthetase (gp29) and gp30 which when expressed together from a prophage confer defense against several unrelated phages including phage Tweety [79]. Expression of Phrann gp29/gp30 strongly inhibits Tweety plaque formation but not Tweety lysogeny. In this case, we propose that Tweety DNA injection results in activation of the (p)ppGpp synthetase, growth arrest, and abortive infection [79].

### A counterdefense system in mycobacteriophage Tweety

Interestingly, Tweety encodes for an intriguing counterdefense system, which is not active against the Phrann gp29/gp30 system but is activatable or “tunable” mutationally [79]. Specifically, Tweety defense escape mutants (DEMs) can be readily isolated that overcome the defense (i.e., form plaques efficiently on a strain expressing Phrann gp29/gp30) and have mutations that map in Tweety gene *54* (Fig 1). Tweety gene *54* is unusual in that it codes for a gp54 protein with unique C- and N-terminal regions flanking 40 to 48 copies of a tetrapeptide repeat of which the first 2 amino acids are alanine [79]. The DEMs vary by the number of these tetrapeptide repeats—either more or fewer—suggesting that specific juxtaposition of the C- and N-terminal “domains” are required for it to be tuned to inactivate the Phrann gp29/gp30 system. We propose a model in which Phrann gp29 normally binds to gp30 to prevent enzymatic activity, and that activation of the system occurs by dissociation of gp29 and gp30 in response to some—as yet unknown—aspect of Tweety infection [79]. However, if Tweety gp54 is tuned to Phrann gp29/gp30 (as in the DEMs), Tweety gp54 may lock in the Phrann gp29/gp30 complex, prevent dissociation, and thus prevent defense.

### Mycobacteriophage genes toxic to bacterial growth

It is likely that phage genes play important roles not just in host range determination, but in other aspects of viral dynamics, including out-competing other phages in lytic growth. Several studies have shown that a substantial proportion (approximately 20%) of phage genes are toxic to the host cell when expressed alone [82,83]. This toxicity itself is likely immaterial to lytic phage growth, as the cell is going to die anyway, and we suggest it more commonly derives from inactivation of cell processes that other phages depend on to lytically infect the bacterium. Thus, a phage undergoing lytic growth can prevent viral “gate-crashers” by shutting down systems that the interlopers are dependent on. One example is Fruitloop gp52, a small (93 amino acids) protein expressed in phage Fruitloop early lytic growth that interacts directly with the host DivIVA protein and inactivates it [82]. An unrelated phage, Rosebush, requires DivIVA to infect, and is therefore excluded from Fruitloop-infected cells by this mechanism [82]. It seems likely that other genes identified as toxic are involved in similar processes [83].

### Phage-based tools for *Mycobacterium* genetics

Mycobacteriophages have facilitated development of several widely used tools for *Mycobacterium* genetics. The slow growth rate and pathogenicity confounded genetic manipulation of *M*. *tuberculosis* for many years, and the need to exploit phage-based systems to overcome these obstacles was recognized early on by Jacobs and colleagues [84]. The extant tools fall into 2 main groups: those that use phage derivatives to take advantage of efficient DNA injection and those that utilize phage genes for tool development. The first category includes phage-based systems for delivery of transposons, allelic exchange modules, and reporter genes and are useful for many *Mycobacterium* hosts, including *M*. *smegmatis*, *M*. *abscessus*, and *M*. *tuberculosis* [85–92]. An obvious advantage of these systems is that phages introduce DNA into bacterial cells at high frequencies, much greater than is typically achieved by transformation or conjugation. Thus, reporter genes can be introduced into clinical samples for rapid determination of drug susceptibilities [88,89,93], transposons can be delivered for the construction of high-density mutant libraries [91], and allelic exchange substrates can be delivered for constructing knockout mutants [85,87]. All of these approaches have been facilitated by the construction and manipulation of shuttle phasmids, which are chimeras that replicate as phages in *Mycobacterium*, and as large plasmids in *E*. *coli* [84,94–96].

Phages have also been used for generalized transduction, as described for phage I3 [97,98], and subsequently for Bxz1 [99] (both in Cluster C), although primarily in *M*. *smegmatis*. Interestingly, all of the phages known to infect *M*. *tuberculosis* have *cos*-packaging systems and are unlikely to mediate generalized transduction. A generalized transducing phage for *M*. *tuberculosis* thus remains a desirable but as yet unachieved goal.

Secondly, several generally applicable tools have been developed from mycobacteriophage genomes. Integration-proficient plasmid vectors have found wide usage, as they facilitate the construction of single-copy recombinants in a wide variety of strains. They simply contain a plasmid vector backbone, a selectable marker, and the integrase gene and *attP* site from a mycobacteriophage; typically, they exclude the phage-encoded RDF, which otherwise would promote plasmid excision. The first such vector was derived from phage L5, which integrates into the *attB*-L5 site (attB-1^smeg^), a short (approximately 40 bp) sequence overlapping a host tRNA^gly^ gene (Fig 3). Transformation with an L5 integration vector is efficient, the host tRNA is “reconstructed” following integration, and recombinants generally are genetically stable [100]; however, they will be lost in the absence of selection if recombinant gene impairs bacterial growth. Excision can be facilitated if desired by introduction of the phage RDF gene [101]. Integration vectors have been described using at least 6 different *attB* sites, including from phages Bxb1 [64], Tweety [32], and Giles [102] (Fig 4A); a multitude of additional vectors could be constructed to expand the suite of useful and compatible plasmids. Because many of the *attB* sites are conserved between *Mycobacterium* species, most of the vectors can be used in both *M*. *tuberculosis* and *M*. *abscessus*, as well as *M*. *smegmatis* (Fig 4). The vectors can also be used with selectable markers based on phage immunity genes rather than antibiotic resistance, using lytic versions of the phages to select for transformants [47,52].

Recombineering systems are also widely used for *Mycobacterium* genetics [103–106]. These mimic the recombineering systems developed from phage lambda and related systems for *E*. *coli* [107,108], which do not work efficiently in *Mycobacterium* strains. Plasmids for *Mycobacterium* recombineering use mycobacteriophage-derived recombinases, most notably from phage Che9c [103–106]. Strains can be readily constructed that express Che9c genes *60* and *61*, encoding an exonuclease and a DNA-pairing protein, respectively, that promote recombination of dsDNA substrates introduced by electroporation [103]. These can be used for constructing gene knockouts and other mutants in both slow- and fast-growing *Mycobacterium* strains. Alternatively, single-stranded DNA (ssDNA) recombineering can be achieved using oligonucleotides, which requires only the DNA-pairing protein (Che9c gp60) [104]. Several important adaptations of these systems are noteworthy, including the ORBIT system that combines ssDNA recombineering with the Bxb1 integration system [109], and its application for the engineering of the phages themselves [110]. This latter system, dubbed Bacteriophage Recombineering of Electroporated DNA (BRED) is a useful approach for constructing phage mutants and recombinants [110–112], which can be made more efficient by coupling with CRISPR-mediated counterselection in the CRISPY-BRED configuration [113].

### Therapeutic applications of mycobacteriophages

The potential therapeutic use of bacteriophages has been postulated for over 100 years, with numerous reports of successful use, especially in eastern Europe and the former Soviet Union [114]. The notion of antimicrobial action by lytic phages is not surprising, especially for topical applications in which access to the pathogen is anticipated, but prospects for lung infections, skin diseases, and disseminated infections such as those caused by pathogenic *Mycobacteria* are much less certain. The first report of phages for treating a *Mycobacterium* infection was for a pediatric cystic fibrosis (CF) patient with a disseminated *M*. *abscessus* infection following a bilateral lung transplant and immunosuppressive therapy to support the new lungs [78]. Resolution of the infection was neither fast nor complete, but there was substantial improvement after about 6 weeks of treatment, with improvement in a large infected node in the liver, reduction in skin nodules, and closure of the sternal transplant wound [78]. There are several important lessons from this case study. First, a considerable search was needed to identify phages that efficiently infect and kill the specific *M*. *abscessus* isolate (designated GD01). Very few phages have been identified using any strain of *M*. *abscessus* as a host for isolation, and de novo phage discovery using *M*. *abscessus* GD01 was mostly unproductive [78]. However, a screen of the large collection of *M*. *smegmatis* phages identified 2 that did, and a third was developed by isolating an HRM of phage BPs. Second, 2 of the 3 phages are temperate, which do not kill bacteria efficiently (see above), and it was necessary to construct lytic derivatives using BRED engineering to remove all or part of the repressor gene [56,78]. Third, the 3-phage cocktail was used with a view to avoid treatment failure due to the emergence of phage resistance. The cross-resistance profiles for the phages were not known, but the 3 phages are genomically distinct (from 3 different clusters), optimizing the prospects for using different infection mechanisms. Indeed, no resistance was observed in any post-treatment isolate. Fourth, the phages were administered intravenously twice daily (10^9^ plaque forming units/dose) without severe adverse reactions. Fifth, no neutralizing antibody response to the phages was observed, although immunosuppressive drugs were being administered [78].

Broadening the use of phages for treating *Mycobacterium* infections must overcome several limitations. Perhaps most importantly, there is substantial variation in the phage infection profiles among clinical isolates of *M*. *abscessus*, and thus the phages useful in the first case are not generally useful for other infections [70]. A screen of 82 clinical isolates illustrates this variation, and also shows that the repertoire of potentially therapeutically useful phages is quite limited (6 to 8) [70]. Moreover, these isolates can be categorized as having either smooth or rough colony morphotypes, and relatively few phages infect and kill any of the smooth strains efficiently. Thus, identifying therapeutically useful phages for *M*. *abscessus* smooth strains is a key priority. Even among rough strains, the phage infection profiles vary considerably among closely related strains [70], and the plasmids and prophages are likely major contributors to the phage infection profiles [51]. About 75% of the rough strains are infected and killed by one or more phages and expanding the repertoire of therapeutically useful phages is also a high priority. Using a broader variety of *M*. *abscessus* isolates for de novo phage isolation may be helpful, as well as exploring the utility of phages isolated on alternative *Mycobacterium* strains. Because *M*. *abscessus* genomes are replete with diverse prophages [51], these may be a good source new phages, following spontaneous induction, growth on a permissive host, and engineering to be obligatorily lytic [70].

Screening of over 200 *M*. *abscessus* clinical isolates facilitated therapeutic interventions in at least 20 additional compassionate use cases of highly drug resistant *Mycobacterium* strains [115]. These revealed a further set of key insights. First, favorable clinical or microbiological outcomes were observed in at least 11 of these cases and are incomplete or inconclusive in 5 others. However, little or no change in clinical outcomes were observed in 4 cases. All of these are confounded by complex circumstances involving other infections and clinical conditions; in any one case the linkage between phage administration and clinical outcome may seem compelling, but the roles of other factors cannot be excluded. For 1 CF patient, phage administration over the course of a year was associated with conversion to sputum culture negative, re-listing for lung transplantation, and an eventual bilateral lung transplant [116]. In another patient, a highly refractory severe *M*. *chelonae* skin infection was resolved after IV administration without any other changes in antibiotic treatment [117]. Interestingly, phage resistance leading to treatment failure was not observed in any of these cases, even though 11 were treated with only a single phage. Although it is premature of conclude that phage resistance is not an impediment to therapy of *Mycobacterium* infections, it may be less of a concern than for some other bacterial pathogens. Finally, emergence of a neutralizing antibody response to the phages was not uncommon. In one case, this correlated with loss of sustained clinical improvement [118,119], but in others, it does not appear to have interrupted clinical improvement. Overall, these compassionate use interventions provide helpful findings for constructing robust clinical trials aimed at elucidating safety, efficacy, dosage, routes of administration, and pharmacokinetics.

Several other *Mycobacterium* diseases might be targets for therapeutic phage treatments including Buruli ulcers caused by *M*. *ulcerans* infections [69,120] and tuberculosis caused by *M*. *tuberculosis* [71,121–124]. Tuberculosis remains an interesting target but presents a different set of challenges than *M*. *abscessus* infections. Importantly, there is much more limited strain variation in the phage infection profiles, reflecting the lack of plasmids and prophages in *M*. *tuberculosis* clinical isolates, such that a small cocktail of phages should be useful for a wide variety of clinical isolates [71]. However, gaining phage access to intracellular bacteria may be a challenge requiring modified delivery systems such as liposomes or “Trojan horse” strategies [125–127], although phage virions can be phagocytosed by macrophages, and transcytosis has been demonstrated [128,129]. However, there could be potential benefits to improving disease and limiting transmission by killing extracellular bacteria, in conjunction with ongoing antibiotic treatments [123]. The prevalence of highly drug resistant strains of *M*. *tuberculosis*, and the threat of resistance to newly developed anti-TB drugs warrants continued investigation into possible roles for phages in controlling this important human disease.

## Conclusions

Our understanding of mycobacteriophages has expanded enormously since they were first described over 70 years ago. However, there is still so much more to learn. We have a high-resolution view of the genomics of phages that infect *M*. *smegmatis*, but there are hundreds of other *Mycobacterium* species and strains that could be used to discover entirely new sets of phages unrelated to these. We note for example, that most *M*. *abscessus* prophages are unrelated to *M*. *smegmatis* phages, and their diversity is at least as great [51]. Preliminary analyses suggest that this is also true for prophages of other *Mycobacterium* strains. We also lack any detailed understanding of the host receptors used for specific recognition by the phages, or more generally what determines host range. It seems likely that there are a multitude of systems contributing to this, including both core bacterial functions and those provided by prophages and plasmids. There are additional genetic tools that could be developed from the phages including in vivo and in vitro packaging systems, vector systems including those using plasmidial prophage origins of replication [45], and phage-based vaccine systems [130]. The therapeutic potential of mycobacteriophages is also in its infancy, and it remains to be seen whether they will prove useful only as treatments of last resort, or whether they are more generally useful. Overall, mycobacteriophages have a rich and promising future.
